# Unique One‐Stage Procedure: Simultaneous Patent Foramen Ovale Closure and Resection of Wilms Tumor With Vena Cava Thrombus in a 5‐Year‐Old

**DOI:** 10.1002/ccr3.73258

**Published:** 2026-07-29

**Authors:** Lea‐Kathrin Hammer, Tsvetomir Loukanov, Patrick Günther, Mina Farag, Angela‐Maria Czundel, Berthold Klein, Markus Kessler, Christoph W. Michalski, Juri Fuchs

**Affiliations:** ^1^ Division of Pediatric Surgery, Department of General, Visceral and Transplantation Surgery Heidelberg University Hospital Heidelberg Germany; ^2^ Division of Pediatric Cardiac Surgery, Department of Cardiac Surgery Heidelberg University Hospital Heidelberg Germany; ^3^ Department of General, Visceral and Transplantation Surgery Heidelberg University Hospital Heidelberg Germany

**Keywords:** cardiopulmonary bypass, inferior vena cava thrombus, patent foramen ovale, pediatric surgery, Wilms tumor

## Abstract

Wilms tumor with intravascular extension poses significant surgical challenges, particularly when combined with a patent foramen ovale, which adds the risk of paradoxical embolism. We report the first case hitherto reported of a 5‐year‐old boy successfully treated in a single‐stage procedure combining surgical PFO closure, nephrectomy, and inferior vena cava thrombectomy. The child recovered uneventfully and remains without complications and tumor‐free at 6‐month follow‐up. This case highlights the importance of thorough preoperative assessment in nephroblastoma patients and demonstrates the value of interdisciplinary collaboration in managing rare and complex pediatric oncologic scenarios.

## Key Clinical Message

This case highlights the first reported one‐stage procedure combining surgical closure of a patent foramen ovale with Wilms tumor resection and inferior vena cava thrombectomy in a child. The approach enabled safe tumor removal and eliminated the risk of paradoxical embolism through interdisciplinary surgical management.

## Graphical abstract

Combined cardiac and abdominal surgery in a child with nephroblastoma, inferior vena cava tumor thrombus extending to the right atrium, and patent foramen ovale.

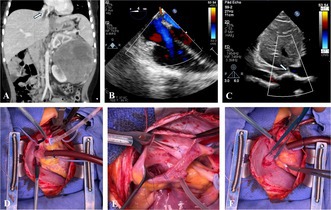

## 1 Introduction

Involvement of the inferior vena cava (IVC) in pediatric nephroblastoma is reported in about 4%–10% of cases, while right atrial extension is even rarer, affecting only 1%–3% of all patients with Wilms tumor [1, 2, 3].

According to the current European treatment protocol (SIOP Umbrella), Wilms tumor with intravascular extension is treated with neoadjuvant chemotherapy, which usually leads to a reduction in tumor size and may reduce the viability of the tumor thrombus [2]. Intravascular extension of nephroblastoma is classified according to Daum et al., and resection strategies must be adapted to the stage [4]. Control of the IVC is required in all cases, with proximal clamping above or below the hepatic veins, depending on thrombus extension. Following IVC clamping, cavotomy or, in case of adherence to the wall, partial cavectomy is performed. In Daum stages I–III, an abdominal approach without cardiopulmonary bypass (CPB) is sufficient. In cases of Daum IV, however, sternotomy with CPB—with or without deep hypothermia and circulatory arrest—is required for complete thrombectomy [2, 3, 4].

The prevalence of patent foramen ovale (PFO) is estimated at 20%–34% [5, 6, 7]. Under circumstances such as the Valsalva maneuver or mechanical ventilation, increased right atrial pressure may induce a right‐to‐left shunt [5, 7]. In the presence of an IVC thrombus, this can lead to paradoxical embolism [5, 6, 7].

While PFO is usually ruled out before chemotherapy in nephroblastoma with atrial thrombus, no cardiac ultrasound had been performed in the present case by the initially treating hospital in the patient's home country. After the tumor was judged unresectable by the local hospital, the 5‐year‐old boy was referred to our center for surgery. As we found no previous literature or reports of simultaneous surgical PFO closure and Wilms tumor surgery with cavotomy and thrombectomy, we present this case of a 5‐year‐old boy undergoing a unique one‐stage procedure.

## 2 Case History / Examination

**FIGURE 1:**
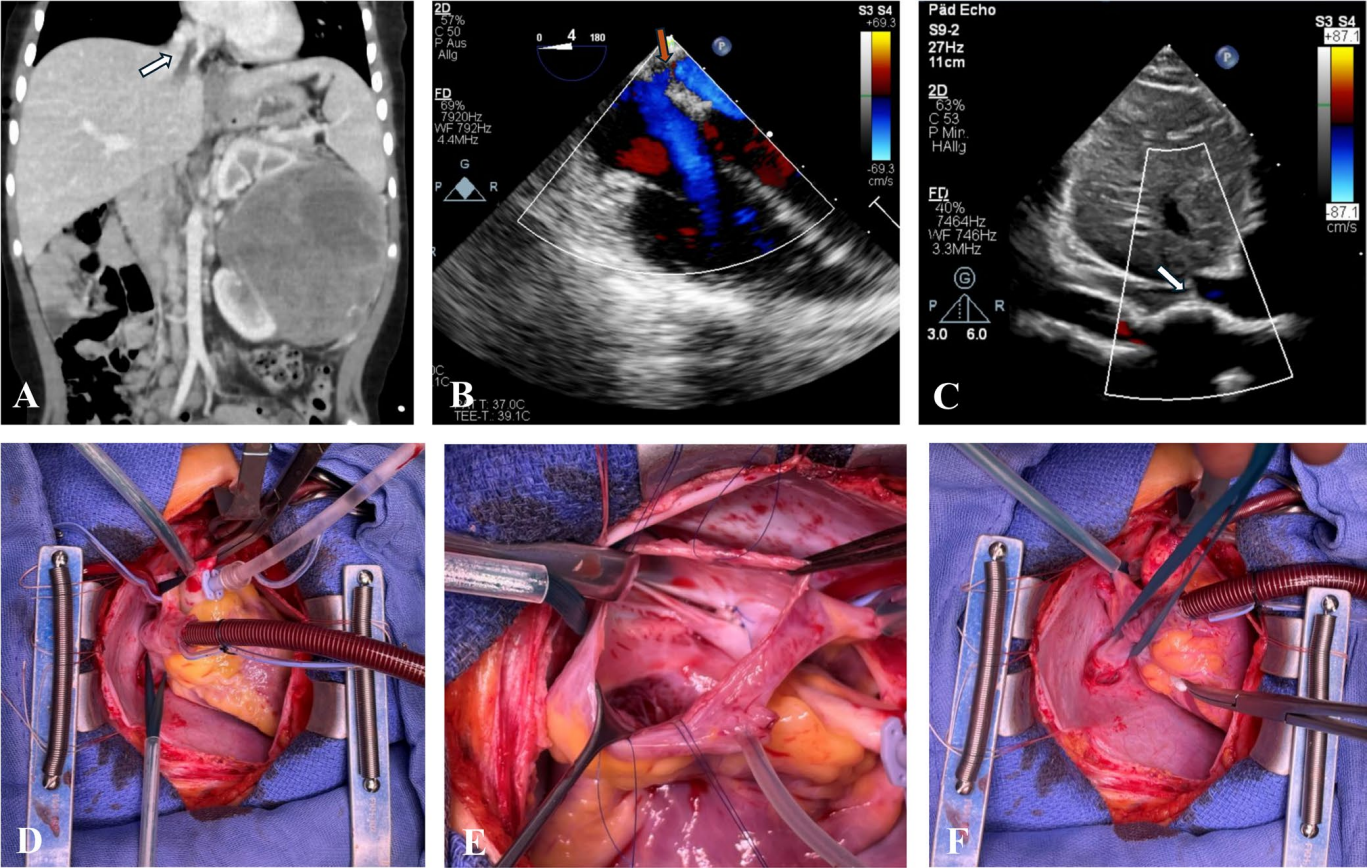
(A) CT‐scan showing the left‐sided nephroblastoma and the inferior vena cava thrombus (white arrow). (B) Transesophageal echocardiography showing the PFO (red arrow). (C) Transesophageal echocardiography showing the inferior vena cava thrombus (white arrow). (D, E, F) Intraoperative views of the PFO closure under cardiopulmonary bypass and circulatory arrest.

A five‐year‐old boy with nephroblastoma of the left kidney and tumor thrombus extending from the IVC to the right atrium presented for surgical assessment in our outpatient clinic. The previously healthy child had first been diagnosed 6 months earlier in his home country after experiencing persistent vomiting for more than 3 weeks. CT scans showed a 12 cm left renal tumor with IVC thrombus extending from the level of the left renal vein to the suprahepatic IVC into the right atrium (Figure 1A). No distant metastases were detected.

He then received chemotherapy as per SIOP‐UMBRELLA protocol for 2 months, which reduced the tumor diameter to about 6 cm. While awaiting surgery in his home country, treatment was paused for 2 months, during which the tumor regrew to 12 cm. The tumor with intravascular extension was then considered unresectable, and 6 further cycles of chemotherapy were administered. Due to progressive symptoms, the patient was referred to our center for a second opinion and surgical evaluation.

## 3 Differential Diagnosis, Investigations and Treatment

Preoperative work‐up at our center included abdominal MRI, abdominal ultrasound, and echocardiography. These revealed limited response of the tumor to chemotherapy, with a diameter of 11 cm, ~500 mL volume, and no evidence of distant metastases. The extension of the tumor thrombus indicated possible involvement of the right atrium. Echocardiography also revealed a PFO with right‐to‐left shunt. The case was discussed in an interdisciplinary team including pediatric cardiology, pediatric oncology, pediatric cardiac surgery, and pediatric oncological surgery. Considering the risk of paradoxical embolism and the need for timely tumor resection without further treatment delay, a decision was made to proceed with a one‐stage operation combining simultaneous oncological surgery and surgical PFO closure by the pediatric cardiac surgery team.

Intraoperative transesophageal echocardiography confirmed the PFO with right‐to‐left shunt and suspicion of thrombotic material in the cavo‐atrial transition (Figure 1B,C). The procedure was then started by the pediatric cardiac surgery team. Following sternotomy and systemic heparinization, cardiopulmonary bypass was initiated and the patient cooled down to 24°C. During 11 min of circulatory arrest, the PFO was closed by direct suture (Figure 1D,E). No thrombotic material was identified within the right atrium. Total CPB time was 85 min including reperfusion. A total of 2 L of allogeneic blood was transfused during the procedure.

**FIGURE 2:**
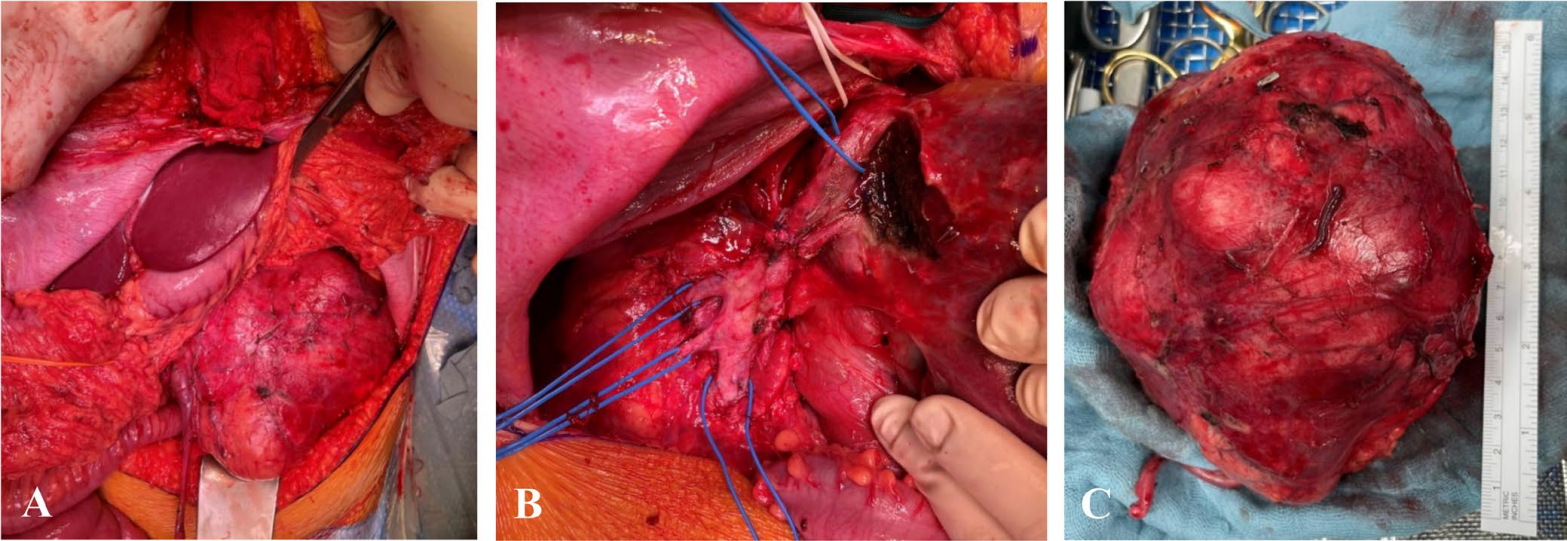
(A) Interaoperative situs after laparotomy showing the large left‐sided nephroblastoma. (B) Situs after tumor resection and thrombectomy showing the inferior vena cava with the right renal veins. (C) Resected nephroblastoma/nephrectomy specimen.

The sternotomy was retained and extended with median laparotomy for abdominal tumor resection. After Cattell‐Braasch and Kocher maneuvers, the IVC, one left, and two right renal veins were exposed. After identifying the superior mesenteric artery, the left renal artery, and the left ureter, left nephrectomy with en‐bloc tumor resection was performed (Figure 2A). No typical intraluminal tumor thrombus was encountered on transection of the left renal vein. The right liver lobe was fully mobilized from the retrohepatic IVC. Intraoperative ultrasound revealed reduced flow and significant wall thickening of the IVC from just cranial to the renal veins up to the hepatic venous confluence. The IVC was briefly clamped below the right renal veins (distally) and just below the hepatic venous confluence (proximally). Cavotomy revealed dense wall thickening with remnants of suspected tumor thrombus, which were removed. Sufficient perfusion of the IVC was confirmed, and cavotomy was closed with a transverse running suture using 5–0 polypropylene (Figure 2B). After interaortocaval lymph node sampling, the abdominal wall and sternotomy were closed. The total operative time (cardiac and abdominal surgery combined) was 8 h and 3 min.

Pathology confirmed completely resected nephroblastoma (R0), blastemal subtype, without lymph node metastases. No viable tumor thrombus was identified, including the IVC wall samples.

## 4 Conclusion and Results (Outcome and Follow‐Up)

Postoperatively, the patient was transferred to the pediatric intensive care unit, extubated after 2 days, and transferred to the normal pediatric surgical ward on postoperative day 8. Postoperative ultrasound of the abdomen and heart showed normal findings. No postoperative complications occurred, and the patient was discharged home after 14 days in excellent physical and neurological condition. Adjuvant chemotherapy and radiotherapy were resumed in the local hospital in the patient's home country. At 6‐month follow‐up, the patient remains in good condition without evidence of tumor recurrence or postoperative sequelae.

## 5 Discussion

In cases with intra‐atrial tumor thrombus, as was suspected in the present patient (Daum stage IV), the use of cardiopulmonary bypass (CPB) and deep hypothermic circulatory arrest (DHCA) is considered the preferred surgical approach. This technique ensures a bloodless field, allowing thorough inspection and removal of the thrombus [2, 3, 4, 8], and CPB would have been indicated in this case regardless of the presence of a PFO. A potential alternative to CPB with DHCA is CPB without circulatory arrest, which may be associated with fewer postoperative neurological complications [9]. It should be noted that the study by Newburger et al. primarily evaluated infants undergoing cardiac surgery. Direct extrapolation to older children, such as our 5‐year‐old patient, must be interpreted with caution, as cerebral tolerance to hypothermia and circulatory arrest may differ with age. Moreover, this approach often requires a higher volume of allogeneic blood transfusions in pediatric oncologic procedures, where intraoperative cell salvage and autologous re‐transfusion remain controversial due to limited evidence regarding oncological safety. Consequently, additional allogeneic transfusions may be required, with attendant risks [10]. Beyond short‐term surgical morbidity, allogeneic blood transfusion has also been associated with adverse oncologic outcomes, including an increased risk of tumor recurrence in pediatric solid tumor patients [10]. This underscores the importance of minimizing transfusion requirements whenever feasible, particularly in oncologic surgery.

PFO closure is generally not indicated in otherwise healthy pediatric patients without associated conditions [6]. When closure is necessary, it is typically performed using percutaneous interventional devices introduced via the femoral vein and the IVC [5, 6]. However, in the presence of thrombotic material within the IVC, this approach carries a high risk of paradoxical embolism and is therefore usually considered contraindicated [11]. Even if the PFO had been diagnosed earlier, percutaneous closure would likely not have been the preferred approach due to this limitation. Preoperative identification of a PFO is essential in patients undergoing cavotomy, as both thrombotic material and potential air emboli can lead to paradoxical embolization through the atrial shunt [3, 12]. If a PFO is detected, aortic cross‐clamping and circulatory arrest are the preferred approaches to safely close the interatrial communication [12].

A critical aspect of this case is the prolonged interruption of preoperative chemotherapy for approximately 2 months prior to referral to our center. According to the current SIOP Umbrella protocol, preoperative chemotherapy in patients with Wilms tumor and intravascular extension is typically administered for 4–6 weeks, followed by surgical resection. The extended pause in systemic therapy was not protocol‐compliant and must be considered oncologically unfavorable. It very likely contributed to the observed tumor regrowth from 6 cm back to 12 cm and may have negatively influenced resectability and overall treatment complexity. We explicitly caution against prolonged treatment interruptions in similar cases, as they may result in tumor regrowth and increased surgical risk.

Analyses of large historic, retrospective cohorts demonstrate that the complete tumor‐thrombus resection significantly impacts event‐free survival in patients with intravascular involvement of Wilms tumor [13]. However, if resection is successful, the overall survival rate is comparable to that of patients without vascular extension, emphasizing the critical role of surgery in these patients [1, 2, 3, 8]. This case is, to our knowledge, the first ever report of a simultaneous surgical PFO closure combined with Wilms tumor resection and cavotomy in a one‐stage procedure. The approach not only enabled safe tumor removal but also eliminated the high risk of paradoxical embolism, underscoring the value of interdisciplinary surgical strategies in complex pediatric oncology cases. Moreover, the case demonstrates the importance of complete staging and diagnostic work‐up in patients with nephroblastoma.

## Author Contributions

**Lea‐Kathrin Hammer:** conceptualization, methodology, validation, writing – original draft, writing – review and editing. **Tsvetomir Loukanov:** conceptualization, methodology, resources, supervision, validation, writing – review and editing. **Patrick Günther:** conceptualization, investigation, project administration, resources, supervision, validation, writing – review and editing. **Mina Farag:** methodology, validation, writing – original draft. **Angela‐Maria Czundel:** validation, writing – review and editing. **Berthold Klein:** methodology, resources, writing – review and editing. **Markus Kessler:** project administration, supervision, writing – review and editing. **Christoph W. Michalski:** resources, supervision, validation, writing – review and editing. **Juri Fuchs:** conceptualization, methodology, project administration, supervision, validation, writing – original draft, writing – review and editing.

## Funding

For the publication fee we acknowledge financial support by Heidelberg University. The authors have no further funding to report.

## Ethics Statement

Additional ethical approval was not required for this single case report in accordance with local regulations.

## Consent

Written informed consent for publication of this case and accompanying images was obtained from the patient's legal guardians.

## Data Availability

All data relevant to this case report are included within the manuscript.

## References

[1] A. Al Diab , N. Hirmas , A. Almousa , et al., “Inferior Vena Cava Involvement in Children With Wilms Tumor,” Pediatric Surgery International 33, no. 5 (2017): 569–573, 10.1007/s00383-016-4034-7. 28070651

[2] D. B. Gehle , Z. D. Morrison , H. F. Halepota , et al., “Wilms Tumor With Vena Caval Intravascular Extension: A Surgical Perspective,” Children (Basel) 11, no. 8 (2024): 896, 10.3390/children11080896. 39201831 PMC11353173

[3] L. Pio , S. Abib , F. Guerin , et al., “Surgical Management of Wilms Tumors With Intravenous Extension: A Multicenter Analysis of Clinical Management With Technical Insights,” Annals of Surgical Oncology 31, no. 7 (2024): 4713–4723, 10.1245/s10434-024-15232-w. 38578552

[4] R. Daum , H. Roth , and Z. Zachariou , “Tumor Infiltration of the Vena Cava in Nephroblastoma,” European Journal of Pediatric Surgery 4, no. 1 (1994): 16–20, 10.1055/s-2008-1066059. 8199126

[5] J. P. Giblett , L. K. Williams , S. Kyranis , L. M. Shapiro , and P. A. Calvert , “Patent Foramen Ovale Closure: State of the Art,” Interventional Cardiology 15 (2020): e15, 10.15420/icr.2019.27. 33318751 PMC7726850

[6] S. Saharan , J. Vettukattil , A. Bhat , et al., “Patent Foramen Ovale in Children: Unique Pediatric Challenges and Lessons Learned From Adult Literature,” Annals of Pediatric Cardiology 15, no. 1 (2022): 44–52, 10.4103/apc.apc_67_21. 35847406 PMC9280096

[7] T. Zhang , C. Gao , W. Chen , H. Ma , and L. Tao , “Patent Foramen Ovale in Children: A Review of Recent Progress,” Pediatric Cardiology 46, no. 5 (2025): 1131–1141, 10.1007/s00246-024-03526-5. 38822852 PMC12021980

[8] Y. Abdullah , J. Karpelowsky , A. Davidson , et al., “Management of Nine Cases of Wilms' Tumour With Intracardiac Extension–A Single Centre Experience,” Journal of Pediatric Surgery 48, no. 2 (2013): 394–399, 10.1016/j.jpedsurg.2012.11.024. 23414872

[9] J. W. Newburger , R. A. Jonas , G. Wernovsky , et al., “A Comparison of the Perioperative Neurologic Effects of Hypothermic Circulatory Arrest Versus Low‐Flow Cardiopulmonary Bypass in Infant Heart Surgery,” New England Journal of Medicine 329, no. 15 (1993): 1057–1064, 10.1056/NEJM199310073291501. 8371727

[10] S. N. Acker , M. M. Nolan , C. Prendergast , et al., “Blood Transfusion Is Associated With Adverse Outcomes in Pediatric Solid Tumor Oncology Patients Following Tumor Resection,” Journal of Pediatric Hematology/Oncology 45, no. 3 (2023): 137–142, 10.1097/MPH.0000000000002530. 36031190

[11] L. He , J. Y. Xue , Y. J. Du , X. G. Xie , X. Y. Wang , and Y. S. Zhang , “Transjugular Approach to Closure of Patent Foramen Ovale Under the Guidance of Fluoroscopy and Transthoracic Echocardiography: A Case Report,” Frontiers in Cardiovascular Medicine 9 (2022): 905614, 10.3389/fcvm.2022.905614. 35669476 PMC9163404

[12] T. Shiratori , T. Fujisawa , T. Ichino , Y. Mitono , M. Inokuti , and J. Ohata , “Anaesthetic Management of a Patient With the Intracardiac Extension of Wilms' Tumour,” Paediatric Anaesthesia 14, no. 4 (2004): 361–364, 10.1046/j.1460-9592.2003.01236.x. 15078385

[13] K. Dzhuma , M. Powis , G. Vujanic , et al., “Surgical Management, Staging, and Outcomes of Wilms Tumours With Intravascular Extension: Results of the IMPORT Study,” Journal of Pediatric Surgery 57, no. 4 (2022): 572–578, 10.1016/j.jpedsurg.2021.08.023. 34565577

